# Early features of autism spectrum disorder: a cross-sectional study

**DOI:** 10.1186/s13052-019-0733-8

**Published:** 2019-11-14

**Authors:** Antonia Parmeggiani, Arianna Corinaldesi, Annio Posar

**Affiliations:** 1 UOC Neuropsichiatria Infantile, Azienda Ospedaliero Universitaria Policlinico S. Orsola-Malpighi, Dipartimento di Scienze Mediche e Chirurgiche, Università di Bologna, Bologna, Italia; 2 Servizio Sanitario Nazionale, Bologna, Italia; 3 IRCCS Istituto delle Scienze Neurologiche di Bologna, UOC Neuropsichiatria Infantile, Bologna, Italia; 4 Dipartimento di Scienze Biomediche e Neuromotorie, Università di Bologna, Bologna, Italia; 5 Ospedale Bellaria, Padiglione G, Via Altura, 3, Bologna, Italia

**Keywords:** Autism spectrum disorder, Children, Early diagnosis, Intellectual disability

## Abstract

**Background:**

Autism spectrum disorder is characterized by impairment in social interaction and communication along with repetitive, restricted, and stereotyped behaviors, interests and activities. It is important to detect this condition as soon as possible and promptly begin targeted treatments. This study aimed to report on age at onset, early signs, and mode at onset in 105 Italian patients with autism spectrum disorder, searching for correlations with a series of clinical and instrumental variables.

**Methods:**

This retrospective cross-sectional study considered the following five categories of symptoms at onset: language, social interaction and relationships, stereotyped behavior and activities, motor skills, and regulation. Three modes of presentation were considered: a delay, a stagnation, or a regression of development, which were defined modes of onset of autism spectrum disorder. The age at onset, the category of clinical features, and the mode at onset were considered in the entire sample and statistically analyzed for several clinical variables. Statistical analysis was performed utilizing Fisher Exact test and Chi Square test.

**Results:**

The first symptoms between 7 and 12 months were evident in 41.9% of cases, and between 13 and 24 months in 27.6%; no significant differences for the age at onset related to diagnosis, etiopathogenesis, early onset epilepsy, and intelligence quotient level emerged. Social interaction and relationships (93.3%) and language (92.4%) were the categories of early signs more represented in our sample. Delay in spoken language (to be understood as both verbal production and verbal comprehension) was one of the most common (even though not specific) symptoms prompting initial medical consultation for a possible diagnosis of autism spectrum disorder. At onset, patients without intellectual disability manifested stagnation more often than delay or regression of development; patients with a severe/profound intellectual disability more frequently showed delay or regression of development. Language signs at onset were less frequent in cases with regression, whereas motor skill disorders prevailed in cases with delay at onset. Feeding problems were more numerous in cases with delay and stagnation of development.

**Conclusions:**

These data contribute to identifying an early trend of autism spectrum disorder, useful also for pediatricians.

## Background

Autism spectrum disorder (ASD) is characterized by impairment in social interaction and communication along with repetitive, restricted, and stereotyped behaviors, interests and activities [1]. The etiopathogenesis is multifactorial, originating from a complex interplay between genetic and environmental factors. In the USA, one in 59 children aged 8 years are diagnosed with ASD [2]; in Italy in our Emilia-Romagna Region in 2016 3.9 cases in 1000 children and adolescents between 0 and 17 years were detected. The mean age of diagnosis is around 4–5 years [3, 4].

It is important to detect an ASD as soon as possible and promptly begin targeted treatments.

Generally, parents worry about the impairment in language and social development that is usually evident during the first 3 years of life; sometimes symptoms are mild and they are recognized later in childhood [1].

In Italy, to promptly recognize ASD, pediatricians are advised to identify early symptoms utilizing the CHAT (Checklist for Autism in Toddlers) [5] beginning at 18–24 months, then if the child’s presentation raises a clinical suspicion of ASD, he/she is evaluated using a specific test (e.g., CARS Childhood Autism Rating Scale and ADOS Autism Diagnostic Observation Schedule) by a childhood neuropsychiatrist, and at this time the definitive diagnosis can be made.

It is noteworthy and at present must be always considered that epilepsy and intellectual disability (ID) or both, may be frequently associated in children with ASD [6–8]. This association may be not casual and it is not sufficiently clear if these three variables (ASD, epilepsy and ID) have a special relationship.

At present, both retrospective and prospective studies have investigated behaviors of ASD children during infancy, informed by parents’ descriptions, early home videos, screening tools and sibling studies [9]. The most common early signs involve joint attention, eye contact, orienting to verbal call, facial expression, social smile and deficit or poor quality of movements. Few studies have investigated autistic features in the neonatal period [10, 11], although some authors have examined preterm infants prospectively [12].

Different data are reported about the age at onset of the early signs: for example, the first decline of social interactions may occur between 2 and 6 months [13]; more generic alterations in sleep, feeding and temperament may occur during the first year in children at risk for ASD [14–17]. The onset mode is characterized sometimes by a regression concerning language and social interaction around 16–20 months [18], sometimes by a psychomotor delay or even by developmental stagnation [19, 20].

At present, retrospective, prospective and review analyses have reached various conclusions. Zwaigenbaum and collaborators underscore that it is important both to consider all the data reported so far and to promote future research [21].

Future research regarding early diagnosis cannot ignore medical comorbidities such as ID, early onset epilepsy and genetic/other medical conditions frequently associated with ASD. For example, it is important to comprehend if, above and beyond any contribution from ID, autism and epilepsy have a relationship.

Our retrospective cross-sectional study on a large population of Italian patients affected by idiopathic (patients without genetic or metabolic diseases or cerebral lesions) and non-idiopathic (patients affected by genetic or metabolic diseases, or with cerebral lesions) ASD aims to describe autism early signs by considering five categories of symptoms at onset. These categories include: a) language, b) social interaction and relationships, c) stereotyped behavior and activities, d) motor skills, and e) regulation.

## Methods

This retrospective cross-sectional study used data from medical records (e.g., medical history, neurologic examination and observation, psychological tests, analysis during hospitalization of home videos recorded by parents). These data, concerning the patients hospitalized at the Autism Centre of the Neurological Clinic of the University of Bologna coming from all over Italy, were collected from February 2001 to July 2011. Parents gave informed consent to the processing of personal data at the time of the clinical evaluation. The study was approved by the Institutional Review Board of the University of Bologna. One hundred and five patients with ASD – 82 males and 23 females (male/female ratio of 3.6:1) – were included.

Forty-six (43.8%) patients out of 105 had a pervasive developmental disorder not otherwise specified (PDDNOS), 4 out of 105 (3.8%) had Asperger disorder, and the remaining 55 cases (52.4%) presented an autistic disorder (AD) according to DSM-IV-TR, 2000 [22]. ASD diagnosis was made utilizing CARS and ADOS. To evaluate the intelligence quotient (IQ) or the development quotient (DQ), depending on the patient age, Wechsler Scales and Stanford-Binet Scale (IQ) or Brunet-Lezine Scale (DQ) were used.

To identify and classify early ASD signs, the following five categories of clinical features were considered following a checklist that was present in each patient’s anamnesis: a) language (e.g., speech not acquired before 3 years, speech delay, regression or stagnation of language, echolalia, comprehension deficit); b) social interaction and relationships (e.g., social interaction with parents/caregivers or peers, joint attention, requesting behavior, reactivity and social orienting, communicative gestures, visual attention to socially meaningful stimuli, social smile, eye contact); c) stereotyped behavior and activities (e.g., hyperkinesia, hypoactivity, stereotypies, atypical object use and atypical games, abnormal sensory interests); d) motor skills (e.g., motor development – fine and gross motor skills, sucking reflex, hypotonia, tonic dialogue); and e) regulation (sleep disorders, feeding disorders: e.g., poor, selective feeding).

As concerns psychomotor development three modes of presentation were considered: a delay, a stagnation, or a regression of development, which were defined modes of onset of ASD.

The age at onset, the category of clinical features, and the mode at onset were considered in the entire sample and statistically analyzed for the following variables: 1) ASD type (PDDNOS and AD), excluding Asperger disorder for a very small number of cases; 2) non-idiopathic (patients affected by genetic or metabolic diseases, or with cerebral lesions) and idiopathic cases (patients without genetic or metabolic diseases, and without cerebral lesions); 3) presence of epilepsy with onset before 3 years of age, because sometimes early-onset epileptic encephalopathy is considered as evidence of the relationship between autism and epilepsy; and 4) presence of an ID.

Statistical analysis was performed utilizing Fisher Exact test and Chi Square test.

We considered significant a *p* value < 0.05.

## Results

### Sample

The mean age at onset of ASD symptoms was 13.6 months (range 3–48 months, median 12 and standard deviation 9.1). In the sample, 57 cases were idiopathic (54.3%), while 48 were non-idiopathic (45.7%)​. The latter presented such conditions as heterogeneous cerebral abnormalities (damages from hypoxic ischemic injury during the pre- and perinatal periods, enlargement of lateral ventricles, incomplete maturation of cerebral white matter, enlargement of the sylvian fissure with moderate temporal-polar atrophy, thinning of corpus callosum, hypoplasia of the cerebellar vermis, polymicrogyria of the right precentral gyrus, Arnold-Chiari malformation type 1, mesial temporal sclerosis, or asymmetry of temporal and frontal horns). Genetic examinations disclosed abnormal high resolution karyotype in 7/105 cases (6.7%): trisomy 21 in two, ring chromosome 22 in one, interstitial deletion of chromosome 2 in one, balanced translocation t (6;10) associated with pericentric inversion of chromosome 9 in one, balanced translocation t (5;10) in one, and pericentric inversion of 7q21.2-7q31.2 in one. Moreover, one patient was diagnosed with Cowden syndrome [23]. Eleven patients (10.5%) had epilepsy with onset before 3 years of age. The awake and sleep electroencephalogram (EEG) recording, performed in all patients, both in those with epilepsy and in those without epileptic seizures, during the follow-up but not at the onset of ASD, excluded in all cases a condition of electrical status epilepticus during slow-wave sleep (ESES). Normal/borderline IQ was present in 19 cases (18.1%), mild/moderate ID in 51 (48.6%), and severe/profound ID in 34 (32.4%). In one patient IQ was not available.

### Age at onset of ASD in the whole sample

Twenty-three cases (21.9%) presented the first symptoms between 0 and 6 months, 44 (41.9%) between 7 and 12 months, 29 (27.6%) between 13 and 24 months, 7 (6.7%) between 25 and 36 months, and 2 (1.9%) between 37 and 51 months.

### Early ASD signs and age at onset in the whole sample, according to the five categories of clinical features

Most patients presented at onset a disorder in the social interaction and relationships (93.3%) and in language category (92.4%), while stereotyped behaviors and activities recurred in 78.1% of the cases, motor skills disorders in 57.1%, and disorders of regulation in 43.8% as feeding disorders and in 32.4% as sleep disorders (Table 1).

**Table 1:** Five categories of early signs: number of cases and percentage

Social interaction and relationships		98/ 105 93.3%	Language	97/ 105 92.4%	Stereotyped behaviors and activities	82/ 105 78.1%	Motor skills	60/ 105 57.1%	Regulation: feeding disorders	46/ 105 43.8%
Abnormal social interaction with peers		80/ 105 76.2%	Language delay	59/ 105 56.2%	Psychomotor agitation/aggression	47/ 105 44.8%	Gross motor developmental delay	46/ 105 43.8%	Selective feeding	27/ 105 25.7%
Abnormal social interaction with parents/caregivers		22/ 105 21.0%	Regression/stagnation	31/ 105 29.5%	Hypoactivity	17/ 105 16.2%	Absence of sucking reflex	17/ 105 16.2%	Scarce feeding	12/ 105 11.4%
Absence of stranger anxiety		29/ 105 27.6%	Echolalia	15/ 105 14.3%	Stereotypies	48/ 105 45.7%	Fine motor development delay	8/ 105 7.6%	Other feeding disorders	25/ 105 23.8%
Lack of social smile		25/ 105 23.8%	Comprehension deficit	54/ 105 51.4%	Atypical games	44/ 105 41.9%	Neonatal hypotonia	9/ 105 8.6%	Regulation: sleep disorders	34/ 105 32.4%
Eye deviation/absence direct gaze		44/ 105 41.9%	Speech not acquired before 3 years	31/ 105 29.5%	Abnormal sensory interests	13/ 105 12.4%	Hypotonia in the first 3 years of age	9/ 105 8.6%		
Joint attention (impairment)		10/ 105 9.5%	Other language abnormalities	20/ 105 19.0%	Psychomotor agitation onset in months (average)	19.9	Tonic dialogue disorder	8/ 105 7.6%		
Absence of response to name		20/ 105 19.0%	Babbling onset months (average)	9.8 (62 patients)	Stereotypies onset in months (average)	24.0				
Reactivity and social orienting (impairment)		34/ 105 32.4%	First word months (average)	20.0 (80 patients)	Hypoactivity onset in months (average)	≤ 24				
Communicative gestures (impairment)	total	36/ 105 34.3%	First phrase months (average)	34.1 (25 patients)						
	pointing	32/ 105 30.5%								
	imitation	7/ 105 6.7%								
Age at onset in months of social interaction and relationship category of early signs (average)		19.7								

### Early signs, age and mode at onset of ASD considering all variables

Considering the main 4 different intervals of age at onset (0–6, 7–12, 13–24, 25–36 months), with the exclusion of 37–51 months because only two cases belonged to this interval, we found no significant differences between AD and PDDNOS, idiopathic and non-idiopathic cases, cases with and without epilepsy starting before 3 years, and among IQ/DQ levels. Only the category of early signs with motor skill disorders was more represented in patients with age at onset between 0 and 6 and 7–12 months (73.9, 64.4% respectively), and the difference was significant (*p* = 0.040).

Table 2 shows all the variables considered, namely ASD type (AD, PDDNOS), non-idiopathic and idiopathic cases, presence of epilepsy before 3 years, ID levels, the 5 categories of early signs, and the age at onset. All these variables were statistically examined (Chi square test) by considering the onset mode (delay, stagnation or regression of the development).

**Table 2:** PDD type, presence of epilepsy before 3 years of age, ID and IQ levels, the 4 categories of early signs, the age at onset

Variables	*P* value	Delay	Stagnation	Regression
AD	*p*=0.047	29/ 48 60.4%	10/ 30 33.3%	16/ 27 59.3%
PDDNOS	*p*=0.093	19/ 48 39.6%	18/ 30 60.0%	9/ 27 33,3%
Non-idiopathic	*p*=0.507	19/ 48 39.6%	15/ 30 50.0%	14/ 27 51.9%
Epilepsy before 3 yrs	*p*=0.605	5/ 48 10.4%	2/ 30 6.7%	4/ 27 14.8%
IQ/DQ				
Normal/borderline	*p*=0.037	6/ 48 12.5%	10/ 30 33.3%	3/ 27 11.1%
Mild/moderate	*p*=0.378	25/ 48 52.1%	16/ 30 53.3%	10/ 27 37.0%
Severe/profound	*p*=0.016	17/ 48 35.4%	4/ 30 13.3%	13/ 27 48.1%
Categories Early signs				
Language	*p*=0.046	46/ 48 95.8%	29/ 30 96.7%	22/ 27 81,5%
Social interaction/relationship	*p*=0.628	45/ 48 93.8%	27/ 30 90.0%	26/ 27 96.3%
Stereotyped behavior and activities	*p*=0.966	38/ 48 79.2%	23/ 30 76.7%	21/ 27 77.8%
Motor skill disorders	*p*=0.0068791	35/ 48 72.9%	15/ 30 50.0%	10/ 27 37.0%
Regulation				
Sleep	*p*=0.830	15/ 48 31.3%	9/ 30 30.0%	10/ 27 37.0%
Feeding	*p*=0.031	24/ 48 50.0%	16/ 30 53.3%	6/ 27 22.2%
Age at onset (months)				
0-6	*p*=0.0054580	15/ 48 31.3%	8/ 30 26.7%	/
7-12	*p*=0.292	24/ 48 50.0%	10/ 30 33.3%	10/ 27 37.0%
13-24	*p*=0.057	9/ 48 18.8%	8/ 30 26.7%	12/ 27 44.4%
25-36	*p*=0.040	/	4/ 30 13.3%	3/ 27 11.1%
37 -51	*p*=0.053	/	/	2/ 27 7.4%

*Abbreviations*: *PDD* pervasive developmental disorder, *ID* intellectual disability, *IQ* intelligence quotient, *AD* autistic disorder, *PDDNOS* pervasive developmental disorder not otherwise specified, *DQ* development quotient

Forty-eight patients out of 105 (45.7%) presented a delay of development as onset mode, 30 (28.6%) stagnation, and 27 (25.7%) regression.

The four cases with Asperger Disorder, being too small as a sample, were not included in the statistical comparison: the mode at onset was stagnation in two of them and regression in the other two. Patients with a developmental delay in 60.4% of the cases had an AD, those with regression in 59.3% had an AD, and those with stagnation in 33.3% had an AD. The difference was statistically significant (*p* = 0.047). There were no significant differences between ASD type, idiopathic and non-idiopathic cases or between patients with and without epilepsy onset before 3 years of age. Cases with normal/borderline IQ/DQ presented stagnation of development at onset more frequently than delay or regression (33.3% vs 12.5% vs 11.1% - *p* = 0.037), while patients with a severe/profound ID presented at onset with a delay or regression of development more frequently than a stagnation (35.4% vs 41.8% vs 13.3% - *p* = 0.016).

Regarding the five categories of early signs, patients with regression at onset manifested language signs less frequently (81.5%) (*p* = 0.046). Motor skill disorders as early signs prevailed in cases with a delay at onset (72.9%) (*p* = 0.0068791). Feeding problems were significantly more frequent (*p* = 0.031) in cases with delay and stagnation of development at onset (50.0 and 53.3%, respectively) than in cases with regression (22.2%). Age at onset between 0 and 6 months was significantly more frequent in the cases showing delay and stagnation of development at onset (*p* = 0.0054580). Age at onset between 13 and 24 months prevailed in the cases with regression of development at onset (44.4%) compared with cases with stagnation (26.7%) and delay (18.8%): this result was near to significance (*p* = 0.057). Age at onset between 25 and 36 months prevailed significantly (*p* = 0.040) in cases with stagnation and regression at onset, while the two cases with age at onset between 37 and 51 months showed a development regression (*p* = 0.053).

## Discussion

Our research considered a large population of Italian patients to identify the ASD early signs and took into account some correlations among variables, which have never been reported.

### Sample and age at onset of symptoms

On account of the extreme etiological variability in ASD, we considered both idiopathic and non-idiopathic cases diagnosed according to DSM-IV-TR criteria [22]. With respect to age at onset of ASD, we found that 41.9% of the cases presented the first symptoms between 7 and 12 months and 27.6% between 13 and 24 months. Notably, there were no significant differences for this variable in relation to the diagnosis according to DSM-IV-TR (see the comparison between AD and PDDNOS), etiology (see the comparison between idiopathic and non-idiopathic cases), early onset epilepsy, and IQ/DQ level. These data confirm that regardless of the etiology or ASD type and the comorbidity with ID and early onset epilepsy, the age at onset may shift more frequently between 7 and 24 months with a peak around 7 and 12 months, even though it is possible to recognize an earlier onset between 0 and 6 months in 21.9% of the cases. In the literature, the data on the age at onset of early signs is extremely variable. The similar age at onset regarding the different ASD types and the IQ/DQ levels were reported also in the review of Daniels and Mandell [24].

Prospective studies are consistent with retrospective studies in finding that for many ASD children, symptoms emerge gradually over the first 18 months or so of life.

We did not consider variables such as higher socioeconomic level, greater parental concern, healthcare, and education, which may influence the age at earlier ASD diagnosis in our study but we wish to underscore the importance of promptly recognizing early signs.

The lack of significant differences for age at onset between AD and PDDNOS indirectly confirms what DSM-5 now takes into account: ASD represents a disorder without the need to consider the subgroups previously reported in DSM-IV-TR.

### Early ASD signs and age at onset in the whole sample according to the five categories of clinical features

Social interaction and relationships (93.3%) and language (92.4%) are the categories of early signs most represented in our sample, which confirms the literature data [20].

Delay in spoken language (intended as both verbal production and verbal comprehension) represents one of the most common (even though not specific) symptoms prompting initial medical consultation for a possible ASD diagnosis.

Categories of stereotyped behavior and activities, motor skills and regulation (feeding/sleep disorders) recurred less frequently (78.1% vs 57.1% vs 43.8/32.4%, respectively); nevertheless, they are equally important. Although repetitive behaviors and interests have been reported in ASD children, little data exist on how these symptoms manifest in early development. In our sample, the most represented subcategories of stereotyped behaviors and activities were stereotypies (45.7%), psychomotor agitation/aggression (44.8%), and atypical games (41.9%). The average age at onset of stereotypies and psychomotor agitation were 24 and 19.9 months, respectively. Hypoactivity recurred in 16.2% of the cases, in 70.6% of which it was evident during the first 24 months of life. Little information is available from retrospective studies, but the literature reports that repetitive behaviors do not appear until after the first birthday [21].

Regarding motor skills, although the subgroup gross motor developmental delay recurred in 43.8% of the cases and it was the most present one, we want to underscore the importance of also finding such conditions as absence of sucking reflex, neonatal hypotonia, hypotonia in the first 3 years of life, fine motor developmental delay, and tonic dialogue disorder early on.

Finally, with regard to the regulation category, feeding problems and sleep disorders may represent a warning early sign suggesting an ASD as it has also been reported in the literature so far [14–17].

In conclusion, our study offers new data on account of the methods we have used (systematic search for correlations among early clinical features and later clinical-instrumental findings) and the consistent sample size we have considered. It is not easy to compare our results with the literature data concerning retrospective studies for methods utilized and sample sizes considered [21, 25]. Larsen and collaborators reported six symptoms differentiating ASD children from typically developing children; this retrospective study examined children who attended day care centers at 12–24 months of age. Our data partly overlap with this study, excluding joint attention impairment which is less frequent in our sample [26]. This discrepancy is perhaps due to the different data source, namely parents in our study, day care personnel in Larsen and collaborators.

### Early signs, age and mode at onset of ASD considering all variables

Our results enrich the literature on the subject with new data; in fact, for the first time, many variables have been considered comparatively.

Regarding the onset age, as we have mentioned above, it is interesting to underscore that there were no significant differences between AD and PDDNOS, idiopathic versus non-idiopathic cases, among IQ/DQ levels, and when epilepsy started before 3 years of age. Once an early diagnosis of ASD is made, it is necessary to do some exams for the etiologic diagnosis for example to distinguish idiopathic and non-idiopathic cases. The identification of a specific condition underlying ASD, as already reported by our group, could be useful to provide medical treatment and to advise family on the ASD recurrence risk [27].

In our sample, AD starts with a developmental delay or regression rather than stagnation (*p* = 0.047). Motor skill disorders were more frequent in patients with age at onset between 0 and 6 and 7–12 months. This finding confirms literature data regarding motor delays and motor impairments that are quite common in ASD [28].

In our study, 60.4% of the patients with delay and 59.3% of the cases with regression as onset modes presented an AD, while only 33.3% of the subjects with stagnation had an AD, the difference was significant (*p* = 0.047). These data, not yet reported in literature, are not easy to explain even if we may suppose that a delay or regression represent something serious more evident in cases with AD rather than in PDDNOS.

Regarding idiopathic and non-idiopathic cases, and patients with and without early onset epilepsy, we found no differences regarding the onset mode. Note that the awake and sleep EEG recording, performed in all patients, both in those with epilepsy and in those without epileptic seizures, during the follow-up excluded in all cases a condition of ESES, which is implicated in the appearance of a heterogeneous spectrum of developmental disorders [29, 30]. However, awake and sleep EEG was not available at the onset of ASD. Only for IQ/DQ, it is relevant to point out that the cases without ID were affected by a stagnation of development at onset more frequently than a delay or a regression (*p* = 0.037). Patients with a severe/profound ID, instead, more frequently had, at onset, a delay or a regression of development (*p* = 0.016). These data are important and suggest that the cognitive level may affect the mode at onset.

With regard to the early signs and their categories, it is interesting to note that language signs were less frequent in cases with regression (81.5%) than in cases with other onset modes (*p* = 0.046); while motor skill disorders as early signs prevailed in cases with a delay at onset (72.9%) (*p* = 0.0068791). Feeding problems were more frequent in cases with delay and stagnation of development at onset than in cases with regression (*p* = 0.031).

All these data, never reported in the literature so far, may contribute to recognizing and tracing an early trend of the pathology.

Age at onset and onset mode seemed to be somewhat correlated with each other: age at onset between 0 and 6 months was significantly more frequent in the cases showing delay and stagnation of development at onset (*p* = 0.0054580), whereas age at onset between 13 and 24 months prevailed in the cases with regression of development at onset (result near to significance: *p* = 0.057). Age at onset between 25 and 36 months prevailed significantly (*p* = 0.040) in cases with stagnation and regression at onset, while the two cases with age at onset between 37 and 51 months showed a regression of development (*p* = 0.053). These last two cases did not have a childhood disintegrative disorder according to DSM-IV-TR [22].

This study presents a few limitations, the most important of which is its retrospective nature. In addition, it was carried out when DSM-IV-TR considered ASD as subdivided into different categories. The sample that we examined may represent a selected population, perhaps more clinically compromised coming to an Italian university centre for Autism. This study did not consider a control population. The sample was examined during a period of 10 years and 6 months, during which the literature increasingly reported data concerning the diagnosis, etiopathogenesis and evolution of autism, leading to significant progress in instrumental examinations: see, for example, the increasingly widespread use of array CGH - Comparative Genomic Hybridization, and secondly of exome sequencing, in the diagnostic work-up of these patients, in view of the important genetic component in the etiology of ASD, also for the purpose of a genetic counseling for the family.

More importantly, this study has several significant strengths. It considers a large sample coming from Italy. In this respect, we wish to underscore the importance of considering the location to improve knowledge of regional variations in age at diagnosis. It consists in an examination of data that were always collected in a consistent way, by the same group of child neuropsychiatrists at the Autism Centre of the University of Bologna, who used the same anamnesis checklist for all the variables considered regarding the early signs. The variables analyzed are completely new. A similar systematic evaluation of data is not reported in the literature and our results contribute to a better definition of the onset mode in relation to the categories of early symptoms, level of IQ/DQ, and presence of early onset epilepsy.

Autism, ID, and epilepsy are highly comorbid, which suggests shared etiologies [7, 31]. In most cases ASD etiology is still a mystery. Recently, Casanova and collaborators studied ID with a known molecular origin by considering ID with high, modest or absent risk of autism or epilepsy, and reported that ID with high rates of ASD comorbidity is present with a particularly homogenous genetic profile. They also reported that genes with high penetrance for syndromic and non-syndromic ASD are localized in the nucleus and are connected in transcription regulation [32]. A correlation among these results and early ASD symptoms is unknown.

## Conclusions

In conclusion our results suggest that: a) early signs in ASD are various and sometimes heterogeneous; b) early symptoms may vary according to the onset mode and age; c) for an early diagnosis of autism, it is not important that a case is idiopathic or non-idiopathic, or is affected by epilepsy early; d) the cognitive level may affect the onset mode.

Our study also suggests that more data regarding early clinical findings and biomarkers are necessary in order to build new strategies for an early ASD diagnosis and thus ensure early treatment and an improved clinical evolution.

## Data Availability

The datasets used and analyzed during the current study are available from the corresponding author on reasonable request.

## Abbreviations

AD: Autistic disorder
ADOS: Autism diagnostic observation schedule
ASD: Autism spectrum disorder
CARS: Childhood autism rating scale
CGH: Comparative genomic hybridization
CHAT: CHecklist for autism in toddlers
DQ: Development quotient
DSM: Diagnostic and statistical manual of mental disorders
EEG: Electroencephalogram
ESES: Electrical status epilepticus during slow-wave sleep
ID: Intellectual disability
IQ: Intelligence quotient
PDD: Pervasive developmental disorder
PDDNOS: Pervasive developmental disorder not otherwise specified
